# Role of Amine Neurotransmitters and Their Receptors in Skin Pigmentation: Therapeutic Implication

**DOI:** 10.3390/ijms22158071

**Published:** 2021-07-28

**Authors:** Enkhmend Enkhtaivan, Chang Hoon Lee

**Affiliations:** College of Pharmacy, Dongguk University, Seoul 04620, Korea; enhmend.1771@gmail.com

**Keywords:** serotonin, histamime, acetylcholine, dopamine, vitiligo, melanogenesis, skin pigment abnormality

## Abstract

Skin pigmentation can occur due to increased melanin, including melanocyte proliferation, melanin biosynthesis, or melanocyte migration. There are many factors that influence the melanin production process, but the role of neurotransmitters in this process is still unclear. We found that histamine and serotonin influence the different stages of melanogenesis and melanogenesis, which increase melanogenesis. Since then, several related papers have been published, and from these papers, it has been recognised that the role of neurotransmitters in skin-pigment-related diseases needs to be summarised. By introducing the role of neurotransmitters in the regulation of various pigment disorders, including vitiligo and melasma, through this review, many researchers can be expected to try to apply neurotransmitter-related agonists and antagonists as treatments for skin pigment disorders.

## 1. Introduction

The skin is the first barrier that separates and protects internal organs from the external environment. Of course, the skin has various functions, including sensory, immune, and nerve systems [1]. Given that skin covers the external surface of the body, it is continually subjected to sunlight, physical impact, and environmental threats such as bacteria [2,3]. Environmental factors are believed to be involved in several skin diseases, including inflammatory skin diseases, benign hyperplastic disorders, hair growth disorders, malignant processes, and pigmentation issues.

One of the critical environmental factors that affect the skin is sunlight. Sunlight is essential for vitamin D synthesis. However, sunlight also causes DNA damage within cells. Therefore, skin cells develop a protective mechanism for DNA through pigmentation. This involves the synthesis of melanin by epidermal melanocytes and its transfer to keratinocytes. This pigmentation can occur in several stages, including melanocyte proliferation, differentiation, melanin synthesis, migration, or dendritic growth [4].

Skin pigmentation is an important concern from the standpoint of human beauty and skin diseases. Many factors influence skin pigmentation [4]. Recently, the neuroendocrine function of melanocytes has been reported by several researchers. For example, melanocytes produce various stress neurotransmitters, neuropeptides, and hormones that are triggered by UV rays, biological factors, and other mediators that play an important role within the neuroendocrine skin system [5,6,7]. While the relationship between skin pigmentation and neural elements, is significant, there do not seem to be many well-established theories relating these to research on skin pigmentation. Therefore, in this review, we examine the role of neurotransmitters and their receptors in skin pigmentation in order to boost the possibility of potentially applying receptor-modulating agonists or antagonists to treat skin pigment disorders.

## 2. Skin Pigmentation

Pigmentation of the skin is related to melanin. It occurs via the transfer of melanin produced from melanocytes to keratinocytes [8,9,10]. This process can be affected by a variety of factors. For example, pregnancy, Addison’s disease, and sun exposure can all make the skin darker [11,12,13,14]. Too little melanin in the body brightens skin. Vitiligo is a disease that causes soft light spots on the skin [15]. Albinism is a genetic condition that affects a person’s skin [16]. People with albinism may have lighter-than-normal skin tones. Infections, blisters, and burns can also make skin lighter [17,18].

### 2.1. Types of Melanin

In humans, melanin is a significant determinant of skin colour. Hair, pigmented tissue under the eye’s iris, and the vascular striatum in the inner ear are sources of melanin. In the brain, melanin-containing tissues include the medulla and pigment-bearing neurons within brainstem regions (such as trajectories). It also occurs in the reticular tissue of the adrenal gland [19,20].

The melanocytes in the basal layer of the epidermis in the skin produce melanin. Humans have similar melanocytes in their skin, but some individuals and ethnic groups have melanocytes that produce varying amounts of melanin. Some humans have little or no melanin synthesis in their bodies; this is known as albinism [16,21].

Different types of melanin differ in the proportions and binding patterns of certain component molecules, i.e., pheomelanin and eumelanin. The latter is the most abundant form of melanin in humans and is the most likely form of deficiency in albinism [22,23].

#### 2.1.1. Eumelanin

Eumelanin polymers have long been thought to consist of polymers consisting of several cross-linked 5,6-dihydroxyindoles (DHI) and 5,6-dihydroxyindole-2-carboxylic acids (DHICA) [24,25]. Eumelanin is divided into brown eumelanin and black eumelanin. These two types of eumelanin have chemically different polymer bonding patterns. Small amounts of black eumelanin without other pigments cause grey hair. Small amounts of brown eumelanin without other pigments cause yellow (blond) hair. As humans get older, the human body continues to produce black eumelanin but stops producing brown eumelanin, resulting in grey hair, which is common in older people [26,27]. Of course, the grey of human hair may occur for other reasons. If you are interested, please see O’Sullivan’s recent review [28].

#### 2.1.2. Pheomelanin

Pheomelanin comes in various colours, from yellow to red [29]. Pheomelanin is particularly concentrated in certain body parts such as the lips, nipples, glans penis, and vagina [30]. When small amounts of brown eumelanin, which can cause blond hair, are mixed with red pheomelanin, the result is orange hair, commonly referred to as “red” or “ginger” hair. As pheomelanin is also present in the skin, redheads often have a more pinkish skin tone. Please note that skin indicating pinkish tone can be attributed to skin vascularisation and blood, especially as a result of inoxification by carboxyhemoglobin following carbon monoxide poisoning. The chemical structure of pheomelanin differs from that of eumelanin in that its oligomeric form contains benzothiazine and benzothiazole units [31,32].

#### 2.1.3. Neuromelanin

Neuromelanin (NM) is a dark polymer pigment found in a population of specific catecholaminergic neurons in the brain. Humans have the highest NM, which is present in small amounts in other primates, but not in many other species [33,34]. Although human NM has been shown to bind efficiently to transition metals such as iron and other potentially toxic molecules, its biological function is still unknown. It may play an essential role in apoptosis and related Parkinson’s disease [35,36]. In addition, trichochrome (formerly called trichosiderin) is a pigment produced in the same metabolic pathways as eumelanin and pheomelanin, but unlike those molecules, it has a lower molecular weight. It occurs in red hair [37,38].

### 2.2. Melanogenesis

#### 2.2.1. Melanocytes

Melanogenesis is defined as the production of melanin pigments. These are most often produced by cells called melanocytes [2,4,8]. Melanocytes are dendritic cells derived from the neuroectoderm [2,5,19,39,40]. Melanoblasts, the premelanocytes, are pigmentless cells derived from embryonic neural crest cells [22,41,42].

After closing the neural tube [23], melanocytes move to various parts of the body and develop into melanocytes and peripheral nervous systems, bone and cartilage in the head, and membrane cells in the eye [2,4,24]. Melanocytes, differentiated from melanoblasts, are found mainly in the skin epidermis and hair follicles [26,29]. Tyrosinase (TYR), tyrosinase-related protein 1 (TRP1), DOPAchrome tautomerase or tyrosinase-related protein-2 (TRP2), premelanosome protein 17 (Pmel 17/gp1000), T cell 1 (MART-1), and microphthalmia-related transcription factor (MITF) are the melanocyte-specific markers.

The melanocytes of the skin are surrounded by keratinocytes (one melanocyte is surrounded by about 30–40 keratinocytes) [31,37,43] and transfer melanin pigments to keratinocytes via direct from melanocytes or keratinocytes [33,37,44]. The molecular structure of melanin is very suitable for the absorption of ultraviolet (UV) and visible light, which prevents ultraviolet radiation (UVR) from sunlight [45,46]. Melanocytes are also found in other body tissues, such as the central nervous and cardiovascular systems, the uvea of the eye, the cochlea and adipose tissue issue [47,48].

**Figure 1:**
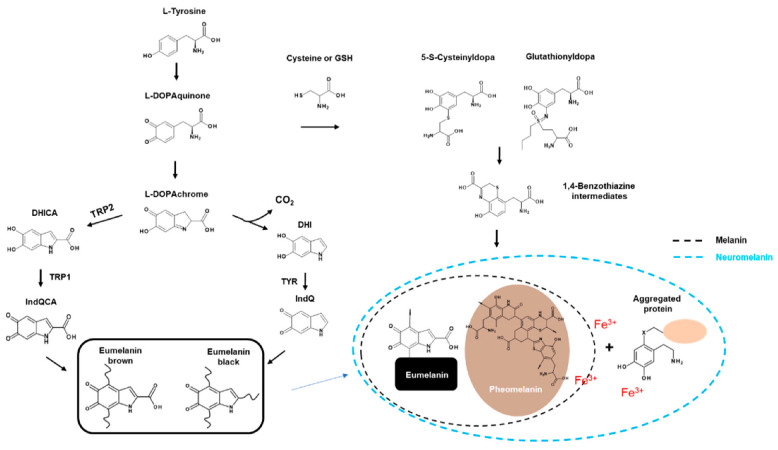
Biosynthesis of melanin.

Melanocytes contain melanosomes, which are organelles similar to lysosomes. Melanin pigments are synthesised and stored before distribution in surrounding keratinocytes [20,49] (Figure 1). Melanosomes require many specific enzymes and structural proteins to mature, which gives them the ability to produce melanin. TYR and TYRP2 are structural proteins where Pmel17 and MART1 are essential for the quantity and quality of melanin [50,51]. AP-3, BLOC-1, and OCA2 play an important role in classifying and trapping melanosomes [52,53,54].

#### 2.2.2. Biosynthesis of Melanin

The most well-known function of melanin is to absorb and scatter UV rays to protect DNA in cells from UV damage. UV irradiation (100–400 nm) is classified into three categories: UVA (320–400 nm), UVB (280–320 nm), and UVC (100–280 nm). The UVC portion of the light spectrum is blocked by the atmosphere’s ozone layer and cannot reach the earth’s surface. Naturally, melanin forms a “cap” or parasol on the nucleus [22,55,56].

Eumelanin is dark and much more polymerised, whereas pheomelanin contains sulphur and is lighter and less polymerised. Eumelanin and pheomelanin come from the same precursor, dopaquinone (DQ). DQ is formed by the oxidation of L-tyrosine through TYR (TYR; Figure 1). The first step in eumelanin production in DQ production involves the spontaneous cyclisation of quinones that produce cyclodopa. This cyclodopa rapidly generates one molecule each of DOPAchrome (DOPAC) and DOPA through a redox exchange with other DQs [26].

DOPAC decomposes through decarboxylation at neutral pH to form DHI and DHICA in a ratio of 70:1 [24]. However, in the presence of TRP2, DOPAC is only converted to DHICA via tautomerisation [29]. Finally, DHI and DHICA are further oxidised and polymerised to form eumelanin (Figure 1).

Pheomelanogenesis consists of several distinct stages at the monomer level. The first step involves the reductive addition of cysteine to DQ to generate 5-S-cysteine Dopa (5SCD) and 2-S-cysteine dopa (2SCD). The second step is the redox exchange reaction between cysteine monodopa (CD) and DQ to produce CD-quinone and DOPA. The third step is CD-quinone cyclisation via dehydration to form ortho-quinonimine (QI). QI is then converted to a 1,4-benzothiazine intermediate with or without decarboxylation. These intermediates are finally polymerised to pheomelanin [30] (Figure 1).

### 2.3. Players in Melanogenesis

The main enzyme involved in synthesising all types of melanin in tyrosine is TYR (EC 1.14.18.1), a single-chain type I membrane glycoprotein [31]. TYR require copper for catalytic function [37]. This oxidase is responsible for the hydroxylation of monophenols to quinone (monophenolase or cresolase activity, EC 1.14.18.1) and DOPA to dopaquinone (diphenolase or catechol oxidase activity, EC 1.10.3.1) in these ortho-diphenols (Figure 1) [15,57,58].

TRP1 and TRP2 belong to the family of Cu^++^/Zn^++^ metalloenzymes that feature a large amount of sequence homology, are expressed in melanocytes, and are mainly localised in unique organelles melanosomes, which play an essential role in melanogenesis. The two proteins share the same signal sequence comprising two cysteine-rich domains and one transmembrane domain [35].

Human TRP1 is encoded by the TRP1 gene (human homologation of the mouse brown gene) found in chromosome 9 (9p23) [45]. A mature form of TRP1, also called gp75, is a protein per 75 kDa membrane transverse [46]. The TRP1 amino acid sequence has a 58 kDa MW. It undergoes glycosylation and acquisition of copper ionic bonds, producing a 17 kDa protein and converting it into a mature active form [22].

The activity of the TRP1/gp75 enzyme in melanogenesis is still quite unclear despite 43% homology with TYR [47]. A series of in vitro biochemical experiments suggested that TRP1/gp75 serves as catalase and weak dihydroxyindolcarboxylic acid oxidase [48,59]. In addition to the unclear enzyme activity, TRP1/gp75 can help stabilise TYR and form xenobiotics in vivo. This complex formation can also weaken cytotoxicity caused by TYR reactions, preventing premature melanocyte death [49].

TRP2, also called dopachrome tautomerase (DCT, EC 5.3.3.12), promotes keto-enol tautomerisation of dopachrome to the relatively more stable intermediate DHICA [29]. In another pathway, spontaneous decarboxylation of dopachrome produces DHI. This metabolic pathway, involving TYR specific to melanocytes, leads to the synthesis of black eumelanin [31,60] (Figure 1).

All three melanin enzymes (TYR, TRP1, and TRP2) that play an important role in melanogenesis are transcription targets of the microphthalmia-associated transcription factor (MITF). The promoters of TYR, TRP1, and TRP2 are activated by MITF. MITF plays a key role in mammalian pigmentation, which is regulated by environmental factors, including UV, and factors secreted by keratinocytes, fibroblasts, and other cells. MITF controls melanogenesis and differentiation, density, proliferation, and cell death through various pathways and mechanisms [4,61].

The activation of PKC-β in the signalling pathway of melanogenesis is involved in regulating TYR activity by phosphorylating serine residues in the cytoplasmic domain of TYR [22].

Several factors released from surrounding cells, such as keratinocytes, fibroblasts, and nerve cells, drive melanin production through receptors. Several receptors have been reported to be involved in melanogenesis. Progress in this area has been extensively reviewed by eminent researchers [4,24,37]. Recent advances are well explained by Park [22].

## 3. Pigmentation Abnormality in Skin Disease

### 3.1. Vitiligo and Albinism

Vitiligo is the loss of melanocytes, resulting in white skin spots. It affects up to 2% of people [15]. The cause of vitiligo is unknown. However, it can be accompanied by an attack of the immune system on the cells that produce skin pigment melanin (melanocytes) [62].

Vitiligo tends to develop either within a family or spontaneously in an individual. Vitiligo can occur with certain other diseases. Vitiligo is associated with autoimmune diseases (when the body attacks its tissues), among which thyroid disease is the most common. It is most closely related to hyperthyroidism (especially when due to Graves’ disease) and hypothyroidism (especially when due to Hashimoto’s thyroiditis) [63]. People with diabetes, Addison’s disease, and pernicious anaemia are also somewhat more likely to develop vitiligo [64]. However, the relationship between these disorders and vitiligo is not clear.

Melanocytes exist in the leptomeninges of the human brain, especially over the ventrolateral surfaces of the medulla oblongata [65]. These leptomeningeal melanocytes also appear to be involved in vitiligo. Aseptic meningitis, observed in Harada syndrome, is likely due to the destruction of leptomeningeal melanocytes [66]. This observation appears to be an interesting example of the presence of a neuro-immune axis in melanocytes.

Oculocutaneous albinism is a rare hereditary disorder in which little or no skin pigment melanin is formed [16]. Only the skin, hair, and eyes, or sometimes only the eyes, are affected [16]. There are four main types of OCA-OCA1 (TYR), OCA2 (OCA2), OCA3 (TRP1), and OCA4 (SLC45A2) [67]. In addition, albinism is observed as a symptom of various genetic diseases. In the case of the WS2 variant of Waardenburg syndrome (WS1-4), the MITF gene exists as a variant, causing albinism [68]. In addition to causing pigmentation of the skin (abnormally small amounts of melanin) or depigmentation (complete loss of pigmentation), it causes decreased vision, misalignment of the eyes (strabismus), and involuntary eye movements (nystagmus) [69]. A type of albinism, called albino, affects the eyes but usually does not affect the skin and hair. A different kind of albinism occurs with bleeding disorders [70].

### 3.2. Hyperpigmentation

Hyperpigmentation is a darkening of the skin, mainly caused by an abnormally large skin pigment melanin [71]. When exposed to sunlight, special skin cells increase melanin, causing the skin to darken or tan. In some people with light skin, certain melanocytes respond to sunlight to produce more melanin than others-this uneven production of melanin results in pigment spots known as freckles. Freckle tendencies occur in families. Factors other than sunlight can increase melanin in spots (topical) or large areas of the skin [72]. In rare cases, the skin darkens because of substances other than melanin.

Local hyperpigmentation can be caused by skin damage, skin inflammation, reaction to sunlight, and abnormal skin growth [73]. Hyperpigmentation can also occur after injuries such as cuts and burns, or inflammation caused by disorders such as acne and lupus [74,75,76].

Hyperpigmentation is often accompanied by other diseases or symptoms. For example, hyperpigmentation can occur in conjunction with blemishes, freckles, black spots, and caffeore spots (flat spots or brown spots), as well as abnormal skin growths such as melanomas [77,78]. People with a disorder called acanthosis nigricans develop dark, thickened skin in the armpits, nape of the neck, and wrinkles of the skin [79]. Melanocytosis can be a symptom of diabetes [80].

Lentigines (commonly referred to as age spots or liver spots (not related to liver problems)) are flat, tan to brown oval spots on the skin [81]. There are two types of lentigines, namely, solar and non-solar lentigines. The solar-type is caused by sun exposure and is the most common type [81]. It occurs most often on the face and the back of the hand exposed to the sun. It usually first appears in middle age and increases in number with age. Lentigines are noncancerous (benign), but people with them may have a higher risk of developing melanomas.

Non-solar lentigines are not due to sun exposure. The non-solar type sometimes occurs in people with certain rare genetic disorders, such as Peutz–Jeghers syndrome (characterised by many black spots on polyps in the lips and intestines), pigmentation, and multiple black spot syndrome (LEOPARD syndrome) [82,83].

Extensive hyperpigmentation can be caused by hormonal changes, internal diseases, drugs, and exposure to heavy metals [19,84]. Hormonal changes can increase melasma in pregnancy or the use of hormonal contraceptives and hyperpigmentation in Addison’s disease [11,85,86]. A liver disease called primary biliary cholangitis (previously called primary biliary cirrhosis) can also increase melanin production [87].

Hyperpigmentation can be caused by drugs, including amiodarone, hydroquinone, antimalarial drugs, tetracycline antibiotics, phenothiazine, cancer chemotherapy drugs, and tricyclic antidepressants. Heavy metals (such as silver, gold and mercury, which can be toxic) can also cause hyperpigmentation [88]. The pigmentation can be purple, bluish-black, tan, or shades of blue, silver and grey, depending on the drug or metal, and is concentrated on the skin [89]. In addition to the skin, the teeth, nails, the whites of the eyes (sclera) and the inside of the mouth (mucous membranes) may be discoloured [90].

## 4. Amines Neurotransmitters and Their Receptors in Skin Pigmentation Diseases

### 4.1. Role of Neurotransmitters and Their Receptors in Melanogenesis

#### 4.1.1. Acetylcholine

**Figure 2:**
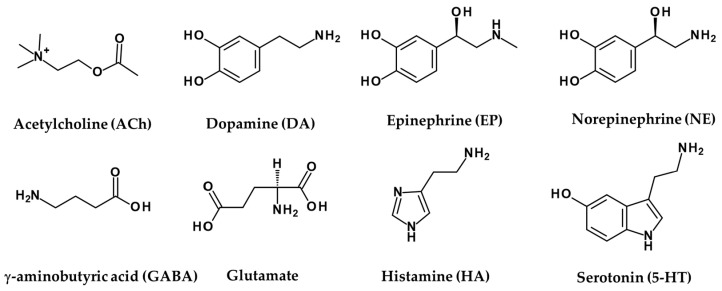
Amine neurotransmitters.

Acetylcholine (ACh) is an organic compound that acts as a neurotransmitter in the brain and body and is usually a chemical message released by nerve cells to signal other cells, such as neurons, muscle cells, and glands (Figure 2) [91]. Acetylcholine receptors (AChR), which recognise and bind the neurotransmitter acetylcholine, are integrated membrane proteins [92].

**Figure 3:**
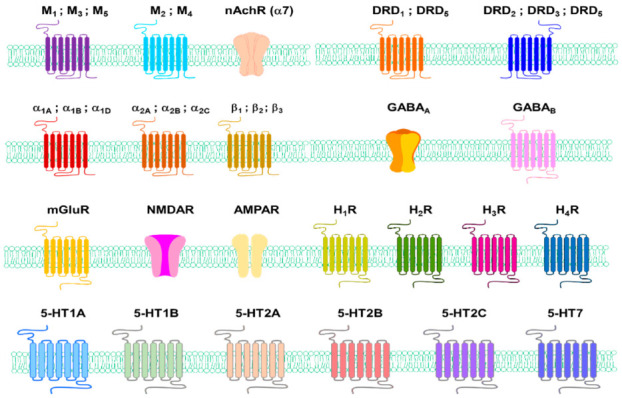
Receptors of amine neurotransmitters.

There are two types of acetylcholine receptors: nicotine (N) and muscarine (M)AChRs. MAChR is located at the neuromuscular junction that causes skeletal muscle contraction through the end-plate potential (EPP). NAChR causes depolarisation in the autonomous ganglia, causing post-neural impulses (Figure 3) [91].

NAChR causes the release of catecholamines from the adrenal medulla and causes site-specific excitation or inhibition in the brain. M and NAChRs are joined by Na^+^ and Ca^2+^ channels, but NAChR is also connected by additional K^+^ channels. NAChRs are ligand–gate ion channels, and the diffusion of Na^+^ and K^+^ across the receptors causes depolarisation, i.e., endplate potentials, opening voltage-gated Na^+^ channels to allow firing of action potentials and, potentially, muscle contraction [91].

AChR, in contrast, belongs to a superfamily of G-protein-binding receptors that activate other ion channels through a second messenger cascade rather than ion channels [93]. MAChRs activate G-proteins when they bind to an extracellular ligand, ACh. G-protein alpha activates the guanylate cyclase (suppressing the effect of intracellular cAMP), while the beta-gamma subunit activates the K^+^ channel to hyperpolarise the cell.

In skin cells, including keratinocytes and MCs, ACh synthesis is catalysed by choline acetyltransferase (ChAT), and degradation is catalysed by acetylcholine esterase (AChE) [94]. The vesicular ACh transporter (VAChT) is responsible for the transport of ACh and choline using a proton electrochemical gradient generated by a vacuole-type H^+^ ATPase and exchanges two luminal protons for one cytoplasmic ACh or choline [95].

Several reports suggest the role of ACh, AChRs, and enzymes related to ACh metabolism in melanogenesis. That is, sunlight, for example, promotes the release of ACh from keratinocytes in the skin, and this increased ACh and AChE inhibitor also suppressed the increased melanogenesis by light [96]. These results suggest the possibility that ACh inhibits melanogenesis and the possibility that AChE may play an important role in the promotion of melanogenesis.

MITFs upregulate AChE expression during melanin production in murine melanoma cells [97]. This report states that AChE is expressed in melanocytes and melanoma cells, and the tetrameric (G4) form is the main AChE isoform in these cells. During melanin production in B16F10 murine melanoma cells, AChE levels decrease significantly. In contrast, ACh stimulates the release of alpha melanocyte-stimulating hormones (α-MSH) from frog pituitary melanotropes through the activation of M and NAChRs [98]. MAChRs mediate nerve-induced pigmentation in free catfish melanophores [99]. The quick changes in skin darkness in lower vertebrates are due to melanosomes concentration or dispersion in the cytoplasm of melanophores, similar to the transfer of melanosomes from melanocytes to the keratinocytes. Despite their neurogenic character, they cannot be considered a response of the melanogenic apparatus to neurotransmitter stimuli, as the net content of melanin does not change.

Normal human skin melanocytes express the MlR, M2R, M3R, M4R, and M5R subtypes of MAChRs in the cell membrane, which controls concentrations of intracellular free Ca^2+^. This can play an essential physiological role in melanocyte behaviour and skin pigmentation [100]. However, there was no result on the role of receptors in melanogenesis in this report. In the following paper, M4R KO mice did not exhibit hair follicle melanogenesis and failed to produce pigmented hair shafts [101]. This suggest that M4R is involved in hair follicle pigmentation.

ACh seems to inhibit melanogenesis, and thus AChE, which induces the degradation of ACh, appears to be involved in the promotion of melanogenesis. Although various receptors mediating the action of ACh exists, there is not much information related to the involvement of these receptors in human melanogenesis. However, M4R seems to be involved in melanogenesis, and M4R is also involved in the pigmentation phenomenon in catfish melanophores, as mentioned above. Therefore, it seems necessary to study the role of AChR in pigmentation. Compared to the results of studies on the role of AChE in pigmentation, the presence of ChAT in melanotrope cells has been reported. Still, its presence in melanocytes does not seem to have been reported [102]. Therefore, the possibility of the existence of ChAT in melanocytes and a study on its role in melanogenesis are also needed.

#### 4.1.2. Dopamine

Dopamine (DA, contraction of 3,4-dihydroxyphenethylamine) is a neurotransmitter that plays several essential roles in the brain and body [103]. Dopamine comprises approximately 80% of the catecholamine content in the brain [104]. Dopamine is an amine synthesised by removing carboxyl groups from molecules of L-DOPA, a precursor chemical synthesised in the brain and kidneys [105].

The actions of dopamine are mediated by five specific cell surface receptors belonging to G-protein-coupled receptor families, D1-like receptors, and D2-like receptors [106]. Dopamine receptors D1 (DRD1) and D5 (DRD5) are members of the D1-like subfamily, while DRD2, DRD3, and DRD4 are members of the D2-like receptor subfamily. G-protein-binding dopamine receptors mediate all the physiological functions of the catecholamine neurotransmitter dopamine, from voluntary exercise and compensation to hormonal control and hypertension [107].

**Figure 4:**
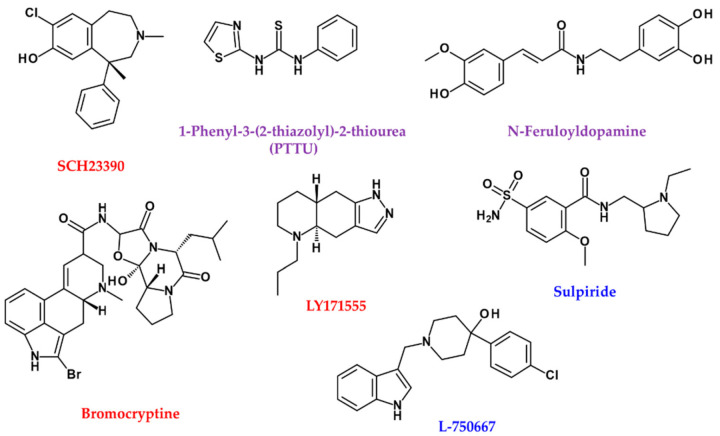
Chemical structures of agonist (red), antagonist (blue), and enzyme inhibitors or others (purple) related to DA neurotransmitters.

The DRD1 and DRD2 are expressed in human skin [108]. α-MSH competes for the action of DRD1 agonists, SCH 23390 (Figure 4) [109]. DA resulted in reduced melanocyte survival at concentrations ranging from 0.01 to 100 M (0.1 and 1 μM, *p* < 0.05; 10 μM, *p* < 0.05; 100 μM, *p* < 0.01). Furthermore, DA-induced melanocyte apoptosis has been demonstrated to increase the ratio of sub-G1 cells from 7.71 ± 0.28% (control) to 12.22 ± 1.05% (0.1 μM DA) (*p* < 0.005) and N-acetylcysteine (NAC), which reverses DA-induced apoptosis [110]. DA treatment induces ROS production, which can be prevented by pre-treatment with NAC. Although DA induces apoptosis of melanocytes, DA can produce melanin in vitro, which is enhanced by pro-oxidant hydrogen peroxide (EC_50_ = 500 μM) and Fe^3+^ but reduced by antioxidants such as ascorbates (IC_50_ = 10 μM) and glutathione (GSH; IC_50_ = 5 μM) [111].

Therefore, derivatives containing agonists and antagonists of DA and its metabolism regulators are also expected to affect melanocytes. While N-nicotinoyl dopamine does not inhibit TYR and melanin synthesis in B16F10 mouse melanoma cells, it inhibits melanosome transfer in normal human melanocyte–keratinocyte co-culture systems [112]. 1-Phenyl-3-(2-thiazolyl)-2-thiourea (PTTU) is a well-characterised dopamine β-hydroxylase inhibitor that suppresses degenerative neurological diseases caused by 6-hydroxydopamine (Figure 4) [113]. Interestingly, PTTU also reduces the enzyme activity and stability of TYR in normal human epidermal melanocytes [113]. N-Feruloyldopamine inhibits human TYR with higher efficacy than arbutin (Figure 4) [114].

Bromocriptine, a dopamine agonist that blocks α-MSH secretion, inhibits melanin production in hair follicular melanocytes of adolescent C3H-HeAvy mice (Figure 4) [115]. Specific DRD2 agonist LY171555 also inhibits TYR activity on the skin’s explants in a dose-related manner, and its effect is blocked by sulpiride, the DRD2 antagonist (Figure 4) [115].

On the other hand, DRD4 antagonist L-750,667, inhibits melanin production through transcriptional downregulation of MITF via ERK signals (Figure 4) [116]. When the melanin precursor molecule DHI (2C) is methylated by catechol-O-methyltransferase (COMT), it cannot be incorporated into melanin [117].

Therefore, the results so far indicate that DA acts in the direction of inhibiting melanogenesis. In particular, although the action of DA receptors has not been verified at the genetic and molecular level, it seems certain that DRD2 activation inhibits melanogenesis and DRD4 activation promotes melanogenesis. Since many compounds with DA moieties affect melanogenesis, it is clear that DA plays an important role in regulating melanogenesis. However, recent research results show that DA can promote melanin production in vitro, but it is not yet confirmed whether this can happen even under intracellular conditions.

#### 4.1.3. Epinephrine and Norepinephrine

Norepinephrine (NE) or epinephrine (EP) is an organic chemical of the family catecholamine that functions as a hormone and neurotransmitter in the brain and body [118]. NE release is the lowest in sleep, increases when awake, and reaches even higher levels during the so-called fight-or-flight reactions in stressful or dangerous situations [119]. EP is produced in NE by N-methylation and is catalysed by phenylethanolamine N-methyltransferase (PNMT) using methyl donor S-adenosyl methionine [120]. EP is usually made by a small number of neurons in the adrenal glands and medulla oblongata. EP plays a vital role in the fight-or-flight response by enhancing blood flow to muscles, increasing heart output by acting on sinoatrial nodes, and increasing pupil dilatation and blood glucose levels [121].

Adrenergic receptors (ARs), or adrenaline receptors, are a type of G-protein-binding receptor targeting many catecholamines, such as NE and EP produced by the body [122]. There are nine ARs: α1A, α1B, α1D, α2A, α2B, α2C, β1, β2, and β3 [123]. Human epidermal melanocytes express α1-ARs and β2-ARs [124,125], autocrine catecholamine biosynthesis and β-adrenergic receptor signals promote pigmentation in human epidermal melanocytes [126]. This paper reports that human melanocytes express functional β2-AR (4230 receptors per cell) of Bmax at 129.3 and 3.19 nM of Kd but lack β1-AR expression. On the other hand, activation of β1-ARs is not affected [124]. β1- and β2-ARs are expressed in tissues of benign melanomas, atypical naevi, and malignant melanomas, and their expression was significantly higher in malignant tumours [127]. The impact of epinephrine on melanin pigment has been observed not only in mammals but in other animals too. An epinephrine-treated frog showed a significant colour change from brown to yellow within 5 min [128]. β-ARs are involved in producing proopiomelanocortin-derived peptides and prolactin induced by histamine in rats [129].

Abundant NE as an exogenous factor causes direct damage to differentiated melanocytes in neural crest cells and suppresses crosstalk between c-kit receptors within melanocytes and the stem cell factor of keratinocytes [130]. NE is secreted into the epidermal microenvironment by nerve endings or keratinocytes, directly inhibiting TYR activity of melanocytes, causing mitochondrial calcium ion malabsorption and free radical production [131].

The secretion of NE due to stress differentiates most of the hair pigment melanocyte stem cells (MeSCs) into melanocytes, leading to the rapid depletion of the pigment-producing cells in the hair follicle to mature melanocytes undergoing apoptosis, and the MeSCs are eventually eliminated. Blocking NE sympathetic nerve system (SNS) signals preserves hair pigmentation in stressed animals. Increased SNS activation has a severe irreversible effect on homeostasis in greying of hair and Alzheimer’s disease [132].

NE seems to act to inhibit melanogenesis and, in the case of EP, to promote melanogenesis, suggesting that PNMT, an enzyme that catalyses the conversion of NE to EP, may influence melanogenesis. However, since this enzyme is not detected well in melanocytes, studies on melanogenesis mainly by PNMT likely require co-culture with keratinocytes [126,133].

#### 4.1.4. Gamma-Aminobutyric Acid

Gamma-aminobutyric acid (GABA) is an inhibitory neurotransmitter that acts on the central nervous system (CNS) of mammals. GABA is responsible for controlling nerve excitation in the nervous system. In humans, GABA directly regulates muscle condition [134]. Although it is difficult to regard it as a neurotransmitter of amines such as dopamine and serotonin (5-HT), GABA must be mentioned in this review because it has an amino group.

GABA receptors are a family of receptors that respond to the neurotransmitter GABA, a major inhibitory neurotransmitter in the mature vertebrate CNS. There are two types of GABA receptors: GABA_A_ and GABA_B_. GABA_A_ receptors are ligand–gate ion channels (also known as ionotropic receptors). GABA_B_ receptors are G-protein-coupled receptors, also called metabotropic receptors. GABA_A_ receptor exists in the epidermal keratinocytes and accelerates cutaneous barrier recovery [135,136]. Although reports of direct expression of GABA receptors in human skin melanocytes have been challenging to find, the action of GABA receptors in melanocytes has been reported in model animals such as larval zebrafish [137]. For example, inhibition of GABA_A_ function, especially GABA_A_ ρ subtype, induces excessive melanocyte population in larval zebrafish [137].

**Figure 5:**
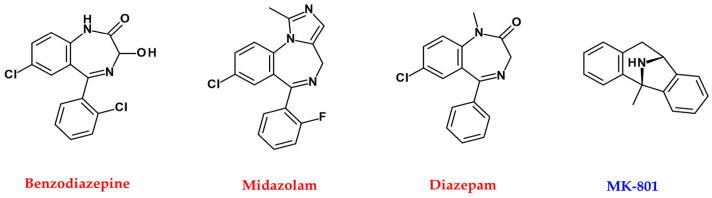
Chemical structures of agonist (red), antagonist (blue), and enzyme inhibitors or others (purple) related to GABA, glutamate neurotransmitters.

By enhancing GABA_A_-mediated anion transport, benzodiazepines depolarise melanoma cells and impair their viability. Benzodiazepine alone reduces tumour growth in vivo and enhances the effect of radiation therapy and α-PD-L1 anti-tumour activity (Figure 5). The combination of benzodiazepine, radiotherapy, and α-PD-L1 results in almost complete regression of treated tumours and a powerful abscopal effect mediated by increased infiltration of multifunctional CD8^+^ T cells [138].

Midazolam prevents cancer metastasis by hyperglycaemia by inhibiting intracellular events and subsequent vascular leakage from the lungs of diabetic mice through GABA_A_ (Figure 5) [139]. Diazepam improves melanogenesis and melanocyte dendritic and melanosome transport through the peripheral benzodiazepine receptor/cAMP/PKA pathway (Figure 5) [140]. Benzodiazepines have high-affinity binding sites and induce melanin production in B16/C3 melanoma cells [141].

It seems that there are not many studies on the effect of GABA on melanogenesis. However, these results suggest that GABA promotes melanogenesis. GABA receptor-related compounds seem to suppress melanoma progression. In the future, studies on which receptors are involved in melanogenesis and how they are involved in melanogenesis seem to have to be verified at the molecular level, beyond the studies using compounds. Additionally, research on how GABA is altered in skin pigmentation disorders and whether related compounds can have therapeutic effects in skin pigmentation disorders may also be needed.

#### 4.1.5. Glutamate

Glutamic acid is one of the typical neurotransmitters in the CNS that primarily acts as an excitatory synaptic, with several types of receptors reported in the nervous system [142]. Glutamate, like GABA, has an amino group, so it was included in this review.

GRM1 is part of the glutamate receptor family, which is divided into two main groups: ionic glutamate receptors (iGluR) and metabolic glutamate receptors (mGluR). mGluR is a seven-membrane spanning-domain G-protein coupled-receptor. The combined receptors are further subdivided into three groups based on sequence-wise and downstream signals. GRM1 and GRM5 belong to groups I mGluR. GRM1 expression was not detected in melanocytes, while GRM5 expression was detected in melanocytes. iGluR contains glutamate–gate ion channels, such as N-methyl-D-aspartate (NMDA) type (NMDAR) or α-amino-3-hydroxy-5-methyl-4- isoxazolepropionic acid type receptors (AMPAR).

GRM1 mediates melanocyte transformation to melanoma through transactivation of insulin-like growth factor 1 receptors [143]. Glutamate receptors such as GRM2 (AMPAR) and NMDAR2A in human melanocytes regulate the expression of MITF [144]. After 24 h treatment with the AMPAR inhibitor CFM-2 at 50 μM, the expression of the key melanocyte differentiation and proliferation factor, MITF, was drastically reduced.

Expression of GRM1 occurs in 60% of human melanomas and cytoplasm but not in benign or normal human melanocytes, suggesting that GRM1 may be involved in melanoma formation [126,127]. Expression of GRM5 promotes melanomas in transgenic mice [145]. GRM6 signalling increases TRPM1 calcium channel function and enhances melanin production in human melanocytes [146]. Stimulation of NMDAR enables filopodia transmission and promotes direct morphological effects on melanocytes to induce melanosome transfer. Whereas 100 μM NMDAR antagonist, MK-801, causes intracellular β-tubulin redistribution and affects the delivery between filopodia between melanocytes and keratinocytes (Figure 5) [147].

Heterotetrameric NMDARs are cationic channels that are primarily permeable to Ca^2+^. The NR1 and NR3 subunits bind to glycine, while the NR2 subunit binds to glutamate for full activation [148]. The NMDAR complex, consisting of NR1-NR3B, is present in the nucleus of melanoma cells. This phenomenon was not observed in melanocytes.

In addition, the NMDAR appears to be involved in the activation of TYR and the promotion of melanin synthesis in the ink gland of *Sepia officinalis*, a cuttlefish [149]. The role of the glutamate transporter rather than the receptor has also been reported. For example, solute carrier family 7 member 11 (SLC7A11) is a cysteine/glutamate exchanger also known as xCT and plays an essential role in synthesising pheomelanin. MITF, MC1R, SLC24A5, Agouti, and CREB1 expression were significantly downregulated after the suppression of SLC7A11 [149]. From these results, it seems clear that glutamate neurotransmitter and related signalling mechanisms are involved in melanogenesis.

#### 4.1.6. Histamine

Histamine (HA) is well known as a substance involved in the local immune response. Still, it also regulates the physiological functions of the intestine and acts as a neurotransmitter for the brain, spinal cord and uterus [150,151].

HA exhibits its biological effects by binding to and activating four different G-protein-coupled receptors (GPCRs) (H_1_R, H_2_R, H_3_R, and H_4_R) [152]. H_1_R and H_2_R exist in keratinocytes and H_1_R in fibroblasts [153]. H_1_R and H_2_R exist in human melanocytes and melanoma cells [154].

**Figure 6:**
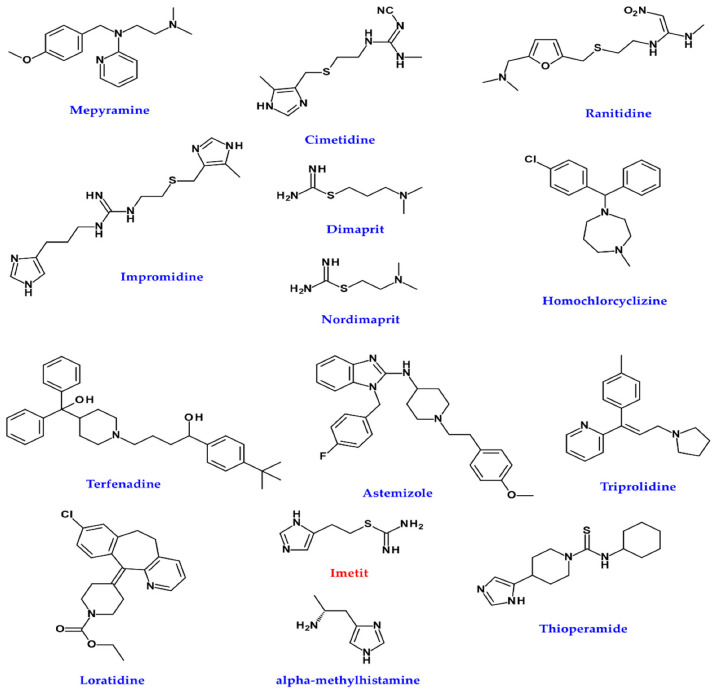
Chemical structures of agonist (red), and antagonist (blue) of histamine.

The possible involvement of HA receptors in melanogenesis has been suggested to take place through the agonists and antagonists of each HA receptor. That is, it was reported that H_1_R antagonist (mepyramine) and H_2_R antagonists (cimetidine, ranitidine, impromidine) increased TYR activity in cultured human melanoma cells, whereas H_2_R agonists (dimaprit, nordimaprit) decreased the activity (Figure 6) [155]. It could be expected that these receptors would inhibit the activation of melanogenesis, but subsequent studies showed opposite results. The H_1_R antagonist homochlorcyclizine (HC) dose-dependently inhibited melanin production in B16 cells stimulated by the α-MSH or 3-isobutyl -1-methylxanthine (IBMX) (Figure 6) [156]. H_1_R antagonists such as terfenadine, astemizole, and triprolidine induce apoptosis in all four melanoma cell lines (Figure 6) [157]. Significantly, H_1_R antagonist therapy did not adversely affect the viability of normal human melanocytes and mouse fibroblasts at the same dose and exposure time [157]. H_1_R antagonist loratadine inhibits melanin production via downregulating MITF in human melanocytes (Figure 6) [158].

Similarly, H_2_R agonists have been reported to decrease the activity of TYR, but HA induces melanogenesis and morphological changes by activating G-protein kinase A through H_2_R in normal human melanocytes [155,159]. HA had a more significant effect on melanocytes proliferation than melanin production [160]. This occurred through H_2_R with complex signals for ERK, CREB, and Akt activation, stimulating melanocyte migration [160]. H_2_R-mediated growth differentiation factor-15 (GDF-15) expression is involved in histamine-induced melanogenesis. Gene silencing of GDF-15 inhibited histamine-induced proliferation, melanogenesis, TYR activity, and chemotropic migration in SK-MEL-2 cells. Histamine-induced expression of TYR, TRP1, and TRP2 was also inhibited by growth differentiation factor-15 gene silencing [41].

TYR was inhibited by the H_3_R agonist imetit but not by alpha-methylhistamine or the H_3_R antagonist thioperamide (Figure 6) [161].

HA partially inhibits proliferation through the stimulation of H_4_R and induces cellular senescence and melanin production in 1205 Lu metastatic melanoma cells expressing H_4_R [162].

According to reports so far, HA receptor-related agonists seem to inhibit TYR activity in vitro, but histamine promotes melanogenesis, and H_1_R, H_2_R, and H_4_R seem to be involved in this process. The possible involvement of H_3_R is still unclear.

#### 4.1.7. Serotonin

Serotonin (5-hydroxytryptamine, 5-HT) is a ubiquitous monoamine that acts as a neurotransmitter in the synapses of neurons [163]. The 5-HT pathway exists in the skin of humans and mice [164,165]. 5-HT itself was detected using immunocytochemistry in immortalised HaCaT keratinocytes and confirmed by reverse-phase high-performance liquid chromatography using the electrochemical detector, which also detects 5-hydroxyindoleacetic acid (5HIAA) [166]. Immunocytochemistry studies of the human scalp have shown 5-HT immunoreactivity in cells of the dermal compartment and epidermal and appendage structures. Significant expression of the 5-HT immune response was detected in cutaneous mast cells. This observation is consistent with the immune detection of 5-HT in perivascular human mast cells in the adrenal cortex [167].

The action of 5-HT is mediated by its interaction with membrane-bound receptors, which can be classified into seven families (5HTR1-7), including at least 21 subtypes [168,169]. Skin cells have 5-HT receptor genes that encode 5-HTR1A, 5-HTR1B, 5-HTR2A, 5-HTR2B, 5-HTR2C and 5-HTR7, and 5-HT has various effects on the proliferation of skin cells (Figure 3) [170]. Several 5-HT receptor (5-HTR) subtypes interact three-dimensionally with the calmodulin via the C-terminal and/or intracellular loops. These interactions can regulate phosphorylation and the consequent desensitisation of the receptor [171].

Studies have shown that these receptors are involved in the physiological function of the skin. For example, the culture of human skin and skin cells expresses mRNA species coding for receptors for 5-HTR1A, 5-HTR1B, 5-HTR2A, 5-HTR2B, 5-HTR2C, and 5-HTR7 [172]. Expression of 5-HTR1A and 5-HTR2A was observed in basal epidermal melanocytes and the epidermis of normal and eczema-like human skin [173]. In addition, 5-HTR3 is expressed in the proliferative base layer of the epidermis [174].

The enzyme tryptophan hydroxylase 1 (TPH1), which catalyses the step of determining the rate of 5-HT synthesis, is expressed throughout human skin. TPH1 transcriptomes of the expected sequence were found in skin samples containing normal skin and basal cell carcinoma, cultured melanocytes, melanoma cell lines, normal keratinocytes, squamous cell carcinoma cells, and fibroblasts (skin and dermal follicles) [175]. Ultraviolet rays (UVR) also inhibit TPH1 expression in squamous cell carcinoma C1-4 cells and human melanoma cells [166].

It was initially reported that 5-HT inhibits melanin production [19]. However, it is not clear which subtype of the 5-HT receptor is involved in this process. The 5-HT concentration used in previous reports was relatively high. Therefore, the effect of 5-HT on melanogenesis has been extensively investigated in three melanocyte-related cell lines, including B16F10, SK-MEL-2, and Melan-a cells [21]. 5-HT increased melanin synthesis levels in B16F10, SK-MEL-2, and Melan-a cells with or without 12-O-tetradecanoylphorbol-13-acetate, which was used as a melanin-producing stimulator in Melan-a cells. 5-HT (100 μM) treated cells showed an increased dendritic network and densely pigmented granules in the cytoplasm. 5-HT dose-dependently increased the migration of B16F10 cells, SK-MEL-2 cells, and Melan-a cells up to four folds compared to untreated cells.

**Figure 7:**
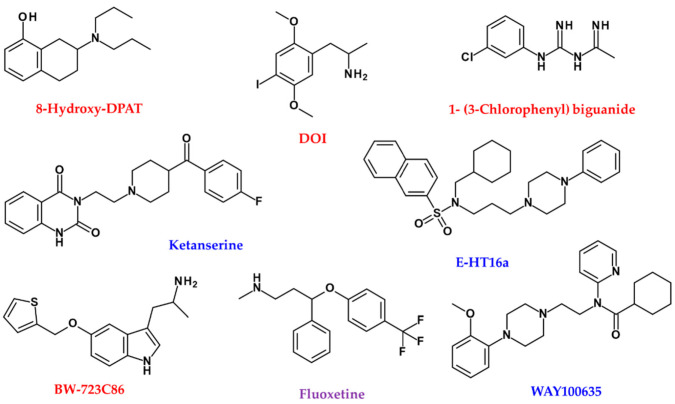
Chemical structures of agonist (red), antagonist (blue), and enzyme inhibitors or others (purple) related to serotonin neurotransmitters.

The effects of 5-HT agonists, including (+)-8-hydroxy-DPAT (5-HTR1A), 2,5-dimethoxy-4-iodoamphetaminehydrochloride (DOI; 5-HTR2A) and 1- (3-chlorophenyl) biguanide hydrochloride (5-HTR3A), on the melanin content of SK-MEL-2 melanoma cells were investigated (Figure 7). DOI, a 5-HTR2A agonist, increased melanin content in SK-MEL-2 cells. DOI increased melanogenesis and TYR activity in SK-MEL-2 cells in a dose-dependent manner. At 72 h after DOI treatment, multipolar branched dendritic networks and dense pigment granules appeared in the cytoplasm of the treated cells in a dose-dependent manner. Chemotactic migration induced by DOI showed a maximum 2.7-fold increase in migrating SK-MEL-2A cells compared to the control.

The 5-HTR2A receptor antagonist ketanserin dose-dependently inhibited 5-HT-induced melanin pigmentation in SK-MEL-2 cells (Figure 7). DOI induced TYR and TRP1 in a dose-dependent manner. The induction of TYR, TRP1 and TRP2 by DOI was blocked by the 5-HTR2A receptor antagonist E-HT16a (Figure 7). Interestingly, 5-HTR2B agonist, BW723C86, decreased melanin synthesis in melan-A cells and human melanocytes (Figure 7) [176]. However, the molecular mechanism of how 5-HTR2B is directly involved in inhibiting melanogenesis remains unknown.

Fluoxetine (2.6 mg/kg, intragastric administration), a famous selective serotonin reuptake inhibitor, improved chronic unpredictable mild stress and chronic restraint stress-induced hypopigmentation in mice, which significantly induced the mRNA and protein levels of the 5-HTR1A and 5-HTR2A receptors in mice and B16F10 cells (Figure 7) [177]. The inducing effects of fluoxetine on the melanogenesis in B16F10 cells and zebrafish were inhibited by WAY100635 (a selective 5-HTR1A antagonist) and ketanserin, respectively (Figure 7) [177]. Activation of the p38 MAPK signalling pathway contributed to fluoxetine-induced melanogenesis and was inhibited by WAY100635, but not ketanserin. However, ketanserin selectively attenuated the ability of fluoxetine to promote migration and up-regulate Rab27a protein expression in B16F10 cells [177]. In particular, R-fluoxetine appears to be important in this effect [178].

There are reports that melatonin derived from 5-HT induces or inhibits melanogenesis [179,180,181,182]. This difference seems to be due to the difference in the concentration of melatonin used and the cell line. In addition, it seems that studies on the possible involvement of melatonin-related receptors have not yet been reported. There are some reports of 5-HT-induced melanogenesis-promoting action, and in particular, 5-HTR1A, 5-HTR1B, and 5-HTR2A seem to be involved in 5-HT-induced melanogenesis [177,178]. On the other hand, 5-HTR2B has the potential to act in the opposite manner.

### 4.2. Role of Amine Neurotransmitters and Their Receptors for Skin Pigmentation Abnormality

In this section, we discuss the effects and possible involvement of the aforementioned neurotransmitters, neurotransmitter derivatives, neurotransmitter-related receptors, and their agonists and antagonists on skin pigmentation disease. In particular, the possibility that neurotransmitters may be involved in skin pigment disease is of great medical significance, because it implies the possibility of treating skin pigment disease using neurotransmitter-related agents and inhibitors. However, it was difficult to find reports on GABA-related skin pigmentation disorders, so GABA-related information was excluded.

#### 4.2.1. ACh

ACh concentration increased with a significant decrease in the expression of AChE in vitiligo patches that return to normal during pigmentation [183]. However, no improvement in vitiligo was observed following treatment with botulinum toxin. AChE activity is reduced in vitiliginous skin [184]. In this study, changes in AChE activity were observed in 52 cases of vitiligo patients during repigmentation and depigmentation. AChE is negative in nondendritic marginal melanocytes during the depigmentation process but positive in pigmentation [184]. ACh can be accumulated during the depigmentation process, e.g., during vitiligo [185].

Mean ACh and H_2_O_2_ levels were significantly higher in vitiligo lesions before NB-UVB (*p* < 0.001), and AChE levels were significantly lower (*p* < 0.001) compared to both repigmented and control skin. In addition, mean MAChR was considerably higher and mean NAChR was substantially lower compared to control and pigmented skin in vitiligo lesions before NB-UVB (*p* < 0.001) [186].

Epithelial H_2_O_2_ in patients with vitiligo has few epidermal AChEs. Molecular modelling based on the established 3D structure of human AChE supports H_2_O_2_-mediated oxidation of Trp (432), Trp (435), and Met (436), moving and redirecting the active site His (440) of the enzyme, leading to protein inactivation [187].

**Figure 8:**
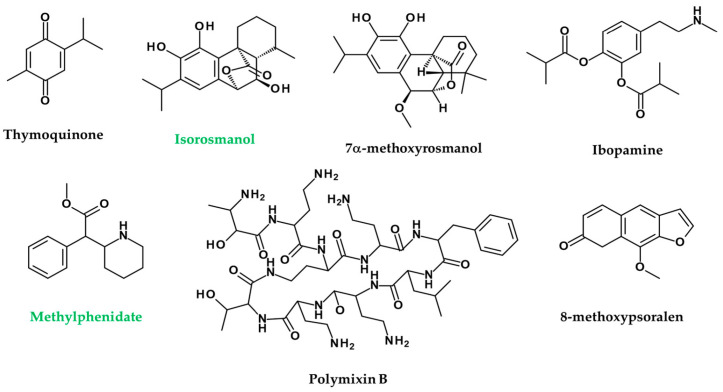
Chemical structures of compounds inducing (black) or inhibiting (green) melanogenesis.

*Nigella sativa* extracts and their active principles mimic the action of ACh in melanin dispersion, darkening the skin through irritation of MAChRs in wall lizards’ melanophores. This study opens up a new prospect, i.e., using the *N. sativa* active ingredient thymoquinone as a new melanogen for clinical application to skin diseases such as low pigmentation and vitiligo (Figure 8) [188].

Seven phenolic diterpenes, namely, sageon, 12-methylcarnosol, carnosol, 7β-methoxyrosmanol, 7α-methoxyrosmanol, isorosmanol, and epirosmanol, are separated from the methanol extracts from the aerial portion of *Salvia officinalis*. Isorosmanol from these compounds showed melanin inhibitory activity as powerful as that of arbutin (Figure 8). Compounds 7α-methoxyrosmanol and isorosmanol suppressed AChE activity by 50% and 65% at concentrations of 500 μM, respectively (Figure 8) [189].

It seems that the role of ACh in inhibiting melanogenesis in vitiligo among skin pigment diseases has been presented. Therefore, substances that lower the level of ACh or inhibit the action of ACh have the potential to improve vitiligo. However, in the case of substances that act on ACh receptors, it seems difficult to apply them widely because the function of the receptors in skin pigment disorders is not yet clear.

#### 4.2.2. DA

There are not many reports on the role of dopamine in abnormal skin pigmentation. Sixty patients with facial vitiligo were divided into two subgroups, i.e., active vitiligo patients (AVPs; *n* = 35) and stable vitiligo patients (SVP; *n* = 25), and 40 healthy subjects were analysed as controls. Plasma levels of DA, including EP, NE, homo-vanillic acid (HVA), 5-hydroxyindoleacetic acid (5-HIAA), 5-HT, and melatonin, showed significant increases in SVP or AVP groups over the control group [190].

COMT is involved in the metabolism of amine neurotransmitters such as DA, EP, and NE, and epidermal homogenates in vitiligo patients showed higher levels of COMT activity than those of healthy controls [117].

The mRNA expression of DRD1 and DRD5, including GPX1, dopa decarboxylase (DDC), and monoamine oxidase A (MAOA), differs in vitiliginous skin, and the protein levels of DRD1, DRD5, DDC, MAOA, and MAOB vary in the skin and/or blood serum of patients with vitiligo [191].

Increased D1-like DRD is observed in unrelated skin and a decrease in affected skin of vitiligo patients [192]. DRD1 levels are decreased in blood sera of vitiligo patients compared with controls [191].

The dopamine-like substance ibopamine caused black deposits on the bandage lens and the front plate of Boston keratoprosthesis, impairing vision (Figure 8) [193].

Methylphenidate is an inhibitor of presynaptic DA and NE transport and effectively treats attention deficit and hyperactivity disorder by increasing DA and NE levels in the synaptic cleft (Figure 8). Cases of leukoderma have been reported following percutaneous methylphenidate patches, in which leukoderma was confined to patch areas, suggesting that vitiligo is directly related to the patches [194].

Protein products from OA1 (GPR143; GPCR, G-protein-coupled receptor), i.e., the ocular albinism type 1 gene, encode pigment cell-specific GPCR confined to melanosomes within the cell, which is also a target gene for MITF [195,196]. Interestingly, GPR143 is a receptor with L-DOPA as a ligand [197].

The action of DA in skin pigment disease was also mainly reported in vitiligo. The expression of DA and DRD1 seems to be increased in vitiligo patients, as DA seems to act in the direction of promoting apoptosis of melanocytes and inhibiting melanogenesis. Therefore, inhibiting the action of DA or blocking the activation of DRD1 in vitiligo patients may promote melanogenesis.

#### 4.2.3. EP and NE

Plasma levels of EP, NE, DA, HVA, 5-HIAA, 5-HT, and melatonin, and serum levels of TSH and prolactin increased significantly in AVPs (*n* = 35) and SVP (*n* = 25) as compared to the control group [190]. Significant increases in plasma and urine catecholamine were observed with vanillylmandelic acid (VMA) compared to healthy controls [198].

Significant increases in the skin α-AR and β-AR responses occurred in segmented vitiligo lesions [199]. However, no changes were found in plasma catecholamine or plasma adrenaline receptor density. These findings suggest that dysfunction of sympathetic nerves occurs in the affected skin and plays a role in causing segmental type vitiligo [199].

EP- and NE-related metabolic enzymes have also been reported to be associated with vitiligo. In other words, epithelial homogenates of patients with vitiligo showed a higher level of COMT activity than healthy controls [117]. The MAOA activity across the entire epidermis in patients with vitiligo increased by 5 to 10 times compared to skin in comparators of matching types. Similar increases in MAOA activity have been found in both established keratinocytes and melanocytes in vitro from the vitiliginous epidermis. Defective catecholamine synthesis in the epidermis in vitiligo patients might increase NE levels with increased MAOA activity [200].

Levels of NE, DA, normetanephrine, metanephrine, 3-methoxy-4-hydroxyphenyl glycol, VMA, and 5-HIAA were significantly higher in patients with progressive vitiligo than in the control group. Progressive vitiligo cases (*n* = 56) showed increased urine excretion values for all parameters (especially NE levels) compared to other patients. Interestingly, in recent cases of vitiligo (<1 year), NE values were different from those of subjects affected in years 1 to 5 and 6 to 10 [201].

Overproduction of (6R) L-erythro-5,6,7,8-tetrahydrobiopterin results in increased plasma and epidermal NE, differentiating keratinocytes, and increasing β-ARs that are defective calcium uptake in keratinocytes and melanocytes [202].

On the basis of these results, EP and NE are closely related to the skin pigment disease called vitiligo. There seem to be few reports on the results of studies on the promotion of melanogenesis through inhibition of EP and NE receptors. That is, although there is a report that geniposide inhibited NE-induced hypopigmentation through GLP-1R-dependent c-kit signalling, this report also did not describe any direct effect in vitiligo patients [130]. Therefore, active research using these EP and NE receptor antagonists or agonists on skin pigmentation disease should be conducted in the future.

#### 4.2.4. Glutamate

As noted earlier, there are not many reports of the direct involvement of glutamate and its receptors in pigment disease, compared to the evidence that glutamate and its receptors are involved in melanogenesis. T helper cell 17 (Th17) has been reported to be potentially linked to the development and progression of vitiligo. In other words, GRM4 regulates the polarisation of T17 cells and alters the melanin production and morphology of B16 cells [203].

Oxidation stress is widely known to contribute to the development of vitiligo. Major transcription factors such as NRF2 (nuclear factor E2-related factor 2) and downstream gene NAD(P)H: quinone oxide-1, γ-glutamyl cystease catalytic subunit, and γ-glutamyl cysteine ligase modified subunit are highly expressed in the lesions of vitiligo patients [204,205].

Antibodies to glutamate decarboxylase (GAD), a rate-limiting enzyme for GABA synthesis, are associated with various autoimmune neurological disorders, including stiff person syndrome, limbic encephalitis, epilepsy, and cerebellar motor dysfunction. Specifically, in a series of 62 patients with anti-GAD antibodies, 63% had dyskinesia, 53% anti-thyroid antibodies, and 16% had vitiligo [206].

Stiff person syndrome is a persistent and painful muscle contraction disorder that mainly affects the axon system, involving autoantibodies to the GAD, which has been associated with diseases such as diabetes, vitiligo, and hypothyroidism. Drugs such as benzodiazepine, which modify central GABA activity, have been used to successfully alleviate symptoms in patients with this syndrome [207,208].

For other indirect examples of involvement, the synaptosomal uptake of glutamate in the visual cortex of albino rabbits was higher than in pigmented rabbits, suggesting that the number of glutamate synapses may be lower in albino rabbits, which in turn seems to indicate differences in glutamate levels and its association with albinos [209].

From these reports, a decrease in glutamate level is likely related to hypopigmentation-related disease, namely vitiligo. Interestingly, agonists and antagonists of glutamate-related receptors have little application to skin pigment disease, so translational studies using these compounds are likely needed in the future.

#### 4.2.5. HA

HA provokes melanogenesis and morphological changes by the formation of cyclic adenosine monophosphate (cAMP), leading to protein kinase A (PKA) activation via HR2 in normal human melanocytes [159]. HR2 is involved in HA-induced melanogenesis [210]. HA stimulates normal human melanocytes in vitro and might be one of the causes of hyperpigmentation in *Urticaria pigmentosa* [182]. Topical application of HA stimulates repigmentation of nonsegmental vitiligo via a receptor-dependent mechanism [211].

Local application of 5% cimetidine in humans increased the melanin index by 38% over five weeks [212]. The administration of polymixin B is known to promote the secretion of HA [213]. Expectedly, 8% of patients with polymixin B application had dermal hyperpigmentation (*n* = 20/249) [214].

Bee venom (BV) stimulates the proliferation and migration of melanocytes, and the production of melanin and HA, a significant component of BV, seems to be an effector through HR2 receptors [160]. Blood HA levels in patients with short periods of vitiligo and itching were significantly increased compared to those in the matching control group [215].

The effect of HA in the blood on melanogenesis in the affected area of vitiligo patients has not been mentioned. Although it has been reported that HC, an H_1_R antagonist, inhibits α-MSH-induced melanogenesis, it is difficult to find case studies related to the treatment of skin hyperpigmentation disease [156]. In the case of HA, the melanogenesis-inducing effect of HA is relatively well-established, so research on skin pigmentation disease treatment using HA agonists and antagonists is likely to be expanded.

#### 4.2.6. 5-HT

Patients with congenital dyschromia showed normal catecholamine metabolism but abnormal tryptophan metabolism, including reduction of 5-HT and melatonin in blood [216]. In patients with Rett syndrome, the 5-HT reduction is associated with melanin reduction in neurons of the substantia nigra [217]. Similarly, all homozygous Hermansky–Pudlak syndrome patients suffer from oculocutaneous albinism and have very low 5-HT content in platelets (15–20% of normal) [218]. Platelets in patients with storage pool disease showed reduced platelet 5-HT levels, and some patients showed albinism [219].

Blood 5-HT levels and anthranilic acid excretion are reduced in patients with vitiligo compared to healthy people [220]. 5-HIAA levels were shown to be high in the early stages of non-segmented vitiligo [221]. This means that 5-HT is converted to 5-HIAA. Regarding treatment, topical or oral use of *Ammi majus* or *Psoralea corylifolia* extract or seeds to supplement vitiligo was known in ancient Egypt, India, China, and Japan, especially when exposed to the sun [222]. Menon of India and El Mofty of Egypt used this as a specific treatment for vitiligo. They use the crystallised active ingredient of *Ammi majus*, mainly 8-methoxypsoralen only, in combination with sunlight or UV exposure [223,224]. The ultraviolet rays and sunlight that were used to treat vitiligo stimulated the skin’s production of 5-HT. In immunohistochemical staining for 5-HT and melatonin, the proportion of 5-HT-positive melanocytes increases with increasing UV exposure, while the proportion of melatonin-positive melanocytes increases with decreasing UV exposure [225]. In addition, the conversion of 5-HT to melatonin is prohibited by UV exposure after 3 h. There was also a low number of melatonin positive melanocytes, and a correspondingly high number of 5-HT positives melanocytes [226].

One of the strongest examples of treatment is interferon-alpha (IFN-α) for hepatitis. Immunotherapy with IFN-α causes significant side effects such as depression and vitiligo. As tryptophan is a precursor to 5-HT, a shortage of tryptophan to the CNS can lead to 5-HT deficiency. Alterations in tryptophan metabolism may contribute to neuropsychiatric side effects of IFN-α [227]. IFN-α induces hypopigmentation by lowering 5-HT, and vitiligo was observed in many case reports using IFN-α therapy against several diseases, including hepatitis [228,229,230,231,232,233,234,235,236,237,238,239]. Thus, our finding that 5-HT is involved in melanogenesis may help explain IFN-α induced vitiligo due to abnormal tryptophan metabolism-induced 5-HT shortage.

Overall, the role of 5-HT in melanin production is well associated with low levels of 5-HT in low-pigmentation diseases such as vitiligo and albinism. However, 5-HT itself, or precursors of 5-HT, may not help treat these conditions, because the metabolism of 5-HT is expected to be significantly higher in these pigment diseases. Therefore, the use of 5-HTR agonists could be an excellent alternative to induce melanogenesis. Additionally, 5-HTR antagonists are also expected to be a good tool for treating skin pigmentation diseases.

## 5. Perspectives

We can find a direct or indirect answer to whether amine neurotransmitters affect melanogenesis, which is a major event of skin pigment diseases. ACh, DA, EP, and NE seem to inhibit melanogenesis, and GABA, glutamate, HA, and 5-HT appear to promote melanogenesis. However, the involvement of the various receptors of each neurotransmitter has not yet been fully elucidated. Therefore, the agonists and antagonists of HA and 5-HT receptors, whose roles in melanogenesis have already been identified, are the most likely candidates for application in the treatment of skin pigmentation disorders.

However, despite these possibilities, there have been few clinical studies using antagonists or agonists of HA and 5-HT for the treatment of skin pigmentation disorders. Additionally, research on the role of each receptor of neurotransmitters such as ACh, DA, EP and NE in skin pigmentation disease seems to be highly insufficient. If it becomes clear which receptors of these neurotransmitters contribute to melanogenesis, clinical applications of agonists and antagonists of these receptors are expected to increase.

Before extending the information obtained from the results so far too clinical studies, it seems that we need to add a few more considerations or studies. First, many studies suggesting the possibility of receptor involvement in melanogenesis and skin diseases have been done using receptor agonists and antagonists. Still, due to possible problems with respect to the selectivity and specificity of these compounds, thorough verification studies using on/off and knockout mice of receptor genes may need to be performed.

Second, of course, the results in melanocytes are more important than the results in melanoma. Still, it is also necessary to check whether the obtained results are the same in melanoma or melanocytes.

Third, it seems necessary to consider the coculture of keratinocytes and melanocytes as a cell system to investigate the effects of neurotransmitter receptors and enzymes of metabolism. We think that this coculture system will better reflect the condition of skin pigmentation disease.

Finally, and this could be the most difficult obstacle, integrated research is needed. In other words, it is necessary to comprehensively study the changes in neurotransmitters and the related signalling machinery in skin pigmentation diseases using methods such as metabolomics, genomics, and proteomics. Of course, it would be ideal if the single-cell-level study was extended in order to better understand which cells are affected by these variations.

Consequently, neurotransmitters are important in the regulation of melanogenesis, and there are already many known drugs that regulate the activity of these neurotransmitters, so we expect to apply these drugs to the treatment of skin pigmentation.

## Data Availability

Not applicable.
